# Monensin Sensitive 1 Regulates Dendritic Arborization in *Drosophila* by Modulating Endocytic Flux

**DOI:** 10.3389/fcell.2019.00145

**Published:** 2019-08-02

**Authors:** Rohit Krishnan Harish, Shweta Tendulkar, Senthilkumar Deivasigamani, Anuradha Ratnaparkhi, Girish S. Ratnaparkhi

**Affiliations:** ^1^Indian Institutes of Science Education and Research, Pune, India; ^2^Agharkar Research Institute, Pune, India

**Keywords:** flux, endocytic recycling, Rab conversion, epistasis, Class IV neuron

## Abstract

Monensin Sensitive 1 (Mon1) is a component of the Mon1:Ccz1 complex that mediates Rab5 to Rab7 conversion in eukaryotic cells by serving as a guanine nucleotide exchange factor for Rab7 during vesicular trafficking. We find that Mon1 activity modulates the complexity of Class IV dendritic arborization (da) neurons during larval development. Loss of Mon1 function leads to an increase in arborization and complexity, while increased expression, leads to reduced arborization. The ability of Mon1 to influence dendritic development is possibly a function of its interactions with Rab family GTPases that are central players in vesicular trafficking. Earlier, these GTPases, specifically Rab1, Rab5, Rab10, and Rab11 have been shown to regulate dendritic arborization. We have conducted genetic epistasis experiments, by modulating the activity of Rab5, Rab7, and Rab11 in da neurons, in *Mon1* mutants, and demonstrate that the ability of Mon1 to regulate arborization is possibly due to its effect on the recycling pathway. Dendritic branching is critical for proper connectivity and physiological function of the neuron. An understanding of regulatory elements, such as Mon1, as demonstrated in our study, is essential to understand neuronal function.

## Introduction

Dendritic arbors are complex neuronal structures with distinct morphological features (Cajal, 1999; Garcia-Lopez et al., 2010; Berry and Nedivi, 2017). During neuronal development, morphogenetic processes that are not yet completely understood, lead to formation of arbors with defined size, geometry, innervation, and tiling patterns. The dendritic tree structure is unique to a given neuronal cell type and plays a fundamental role in establishing specific neuronal connectivity. An intrinsic genetic program patterns the arbors using molecular processes that are distinct from those that make axons. These are found to be dependent on both, internal as well as external cues (Parrish et al., 2007; Jan and Jan, 2010). The growth and development of dendritic arbors are also concurrent in time and space with synapse formation with proteins of the post-synaptic density playing an integral role in morphogenesis (Cantallops et al., 2000; Cline, 2001; Peng et al., 2009).

The embryonic and larval peripheral nervous system (PNS) in *Drosophila melanogaster* has served as an excellent model system for studying mechanisms that govern dendritic arbor complexity and tiling. The PNS consists of 45 sensory neurons per hemisegment which are classified into type I and type II neurons (Grueber et al., 2003; Orgogozo and Grueber, 2005). The type II neurons are multidendritic whose dendrites innervate the epidermis. Dendritic arborization (da) neurons are a type of multidendritic neurons which are further classified into class I to IV on the basis of their dendrite field complexity with class IV da neurons having the most complexity in terms of the number of dendrites and their branching (Grueber et al., 2002). The arbor complexity in da neurons is determined through a combinatorial expression of transcription factors indicating the process is hard-wired and intrinsic to the neuronal class (Jinushi-Nakao et al., 2007).

As in other organisms (Dong et al., 2015; Prigge and Kay, 2018) the process of morphogenesis is regulated by signaling mediated by external cues such as Slit and Semaphorins (Jan and Jan, 2010; Meltzer et al., 2016), kinases such as Tricornered (Emoto et al., 2004) and a range of cellular processes that include intracellular trafficking, translational control and cytoskeletal dynamics (Ye et al., 2004; Satoh et al., 2008; Delandre et al., 2016).

Rab proteins are key regulators of intracellular trafficking. Both endocytic and exocytic pathways are believed to contribute to dendrite growth and branching (Jan and Jan, 2010; Dong et al., 2015; Valnegri et al., 2015). Constituents implicated include Rab5 (Satoh et al., 2008; Mochizuki et al., 2011; Copf, 2014; Zhang et al., 2014; Kanamori et al., 2015; Wang et al., 2017), Rab10 (Zou et al., 2016), Shrub (Sweeney et al., 2006), and Rop (Peng et al., 2015).

In this study we demonstrate that *Drosophila* Monensin Sensitivity 1 (DMon1; Yousefian et al., 2013; Deivasigamani et al., 2015; Dhiman et al., 2019), a core component of the Mon1:CCZ1 complex (Wang et al., 2002; Nordmann et al., 2010; Poteryaev et al., 2010), and central to conversion of early endosomes to late endosomes, regulates morphogenesis of Class IV da (CIVda) neurons. We uncover a role for Mon1 by demonstrating that CIVda patterning can be regulated by increasing or decreasing Mon1 function during embryonic/larval development: loss of Mon1 leads to increased branching while overexpression suppresses it. Consistent with its position in the endocytic pathway, we find that Mon1 functions genetically downstream of Rab5. Surprisingly however, the regulation by Mon1 does not seem to be dependent on the late endosomal-lysosomal pathway. Rather, the modulation appears to be via the Rab11 mediated recycling pathway. We propose that in the context of the da neurons, Mon1 serves to balance the endocytic flux flowing through the endo-lysosmal and recycling pathways to regulate dendrite morphogenesis.

## Results

### Mon 1 Modulates Dendritic Branching in Class IV da Neurons

**FIGURE 1:**
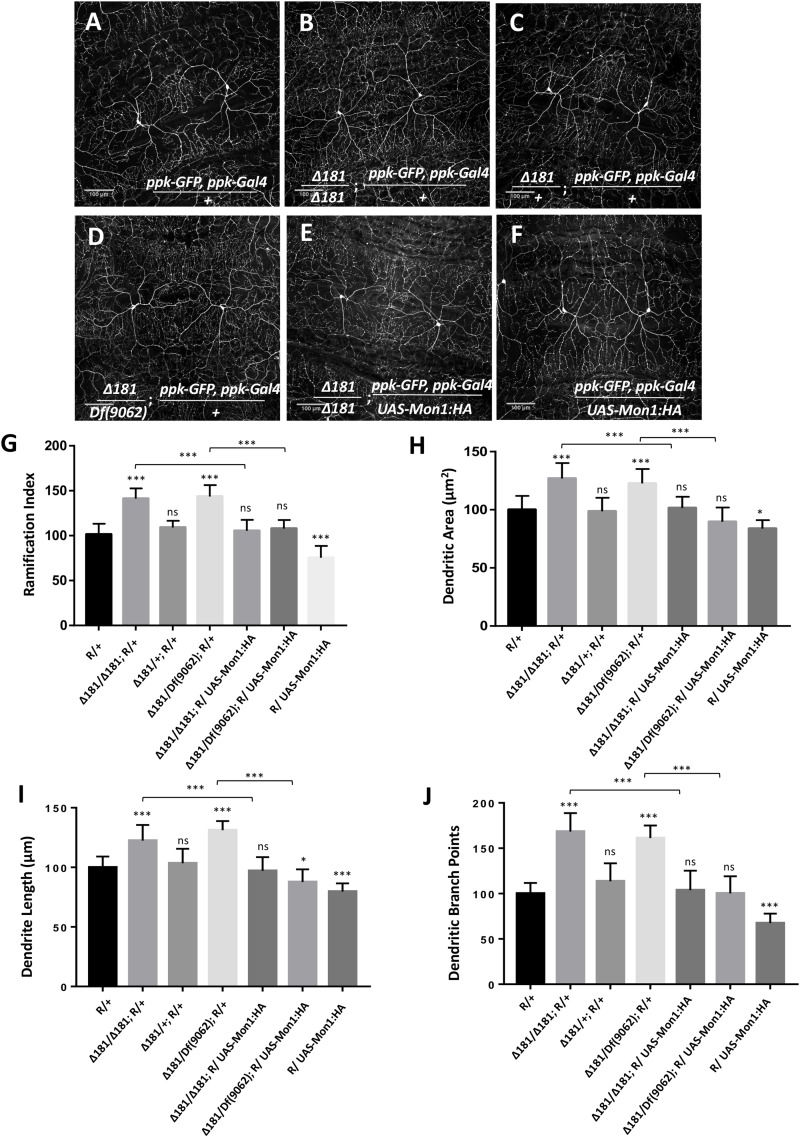
*Dmon1* modulates dendritic arborization in Class IV da -neurons. **(A)** A reporter (*ppk-Gal4; ppk-GFP*, diminutive ‘R’) line is used to visualize arborization in CIVda neurons at the third instar larval stage in *Drosophila melanogaster*. Sholl analysis (Image J) is used to calculate Ramification Index (R.I), which is then normalized, setting the ‘R/+’ at 100. The IMARIS software is used for neuron tracing and calculation of Dentritic area (D.A), Dendrite length (D.L) and Dendritic branch points (D.BP), with each parameter for the R/+ set at 100. *n* = 15 (neurons), *N* = 4 animals. Representative images are shown for this and other images **(B–F)**. **(B)** *Dmon1^Δ181^/Dmon1^Δ181^* larvae show enhanced arborization for Class IV arbors. *n* = 15, *N* = 4. **(C)** A Single copy of *Dmon1* (*Dmon1^Δ181^/+*) does not show any significant increase in arborization. *n* = 14 neurons, *N* = 4. **(D)** *Dmon1^Δ181^/Df(9062)* increases arborization to the same extent as *Dmon1^Δ181^/Dmon1^Δ181^*. *n* = 15, *N* = 4. **(E)** *UAS-Mon1:HA* driven by *ppk-Gal4* in *Dmon1 ^Δ181^* larvae, rescues the arborization defect in *Dmon1^Δ181^/Dmon1^Δ181^* and *Dmon1^Δ181^/Df(9062)* (Image not displayed) to near normal levels. *n* = 18, *N* = 5 and *n* = 9, *N* = 4 respectively. **(F)** Overexpression of *Dmon1* in a wild-type background, reduces the branching (D.BP), R.I and D.L significantly. The reduction in D.A is less significant. *n* = 35, *N* = 8. **(G–J)** Quantitation of the extent of arborization in CIVDa using four parameters, R.I, D.A, D.L, and D.BP. Statistical analysis using Dunnet’s multiple comparison test using GraphPad Prism 7 with exact *p*-values listed in Supplementary Table S1. ns, not significant. ^*^p < 0.05 and ^∗∗∗^*p* < 0.001. Error bars represent standard error.

CIVda neurons express *pickpocket* (*ppk*), a gene involved in nociception in *Drosophila* (Adams et al., 1998; Crozatier and Vincent, 2008). We recombined *ppk*-*Gal4* (BL32079; Grueber et al., 2007; Kanamori et al., 2013) with a membrane localized GFP expressed under a *ppk* regulatory element (*ppk*-*GFP* (BL35843) (Kanamori et al., 2013), and generated a reporter line (‘R,’ see section “Materials and Methods”) that allows visualization of CIVda neuron morphology in response to genetic manipulation either through gene knock-down and overexpression in the third instar larva of *Drosophila* (Figure 1A). This reporter (‘R’) line was used to observe the arborization of CIVda in the *Dmon1*^Δ181^ (Δ181) line, a loss of function allele of *Mon1* (Deivasigamani et al., 2015). When compared to a wild-type control, CIVda in *Dmon1* mutant showed enhanced dendritic branching with a ramification index (R.I), of approximately** 80 as compared to 60 in wild-type larvae. On normalization, with the reporter line set to 100, *Dmon1* mutant shows 45% increase in R.I (Figures 1B,G). This increase in R.I was however not observed in a heterozygous condition (Figures 1C,G), suggesting that a single copy of *Dmon1* is sufficient to regulate dendritic arborization in the CIVda neurons. In order to confirm the result, we quantified R.I in *Dmon1^Δ181^/Df(9062)*, an allelic combination, where Df(9062) is a deficiency that uncovers the *Mon1* locus (Deivasigamani et al., 2015). An increase in RI by 47% (Figure 1G) similar to that in *Dmon1*^Δ181^ homozygote confirmed that it is the loss of *Dmon1* that leads increased R.I of CIVda neurons. Other parameters (see section “Materials and Methods”), such as dendritic area (D.A; μM^2^), dendritic length (D.L; μM) and number of dendritic branch points (D.BP) were also measured for the same set of images. Loss of *Dmon1* also led to an increase in average values, as compared to controls for D.A (51843 vs. 65787 μM^2^), D.L (14549 vs. 17811 μM) and D.BP (492 vs. 829). Normalized values, with the control R/+ set to 100 are displayed in the figures (Figures 1H–J).

Further confirmation for the role for Mon1 in regulating CIVda branching was demonstrated by rescue of the dendritic phenotypes in homozygous *Dmon1*^Δ181^ (Figure 1E) and *Dmon1^Δ181^/* Df(9062) animals through expression of DMon1 in the *ppk* domain (Figure 1G). In both examples, the R.I, D.A, D.L, and D.BP were restored to wild-type or near wild-type levels (Figure 1).

In addition, overexpression of Mon1 in wild-type animals using *ppk-GAL4* led to reduction of all four parameters measured (Figure 1). R.I, D.L, and D.BP were reduced significantly, while the reduction of D.A had lower statistical significance (‘^*^’; Figure 1H). Together, these results demonstrate that arborization of the CIVda neurons during development is sensitive to the dose of Mon1 with decrease in Mon1 function leading to increased branching, dendritic length and area while enhancement of Mon1 function leads to a decrease in the measured parameters.

### Rabs Modulate Dendritic Arborization

Mon1/SAND1 regulates Rab conversion in yeast, *C. elegans* and mammalian cells (Nordmann et al., 2010; Poteryaev et al., 2010; Yousefian et al., 2013). Mon1 in complex with Ccz1 functions as a guanine nucleotide exchange factor for Ypt7, the yeast ortholog of Rab7 (Nordmann et al., 2010). As in other model systems, in *Drosophila*, the recruitment of Rab7 on late endosomes is mediated by the Mon1-Ccz1 complex (Yousefian et al., 2013). In the study by Yousefian et al. (2013), Mon1 loss of function leads to enlargement/enrichment of Rab5 positive early endosomes and concomitant loss of association of mature endosomes with Rab7, a feature that is replicated in CIVda neurons (Supplementary Figure S1). Rab4 and Rab5 co-localized on early endosomes while Rab11 distributions between mutant and wild-type cells were indistinguishable (Yousefian et al., 2013), a feature seen here in CIVda neurons (Supplementary Figure S1).

**FIGURE 2:**
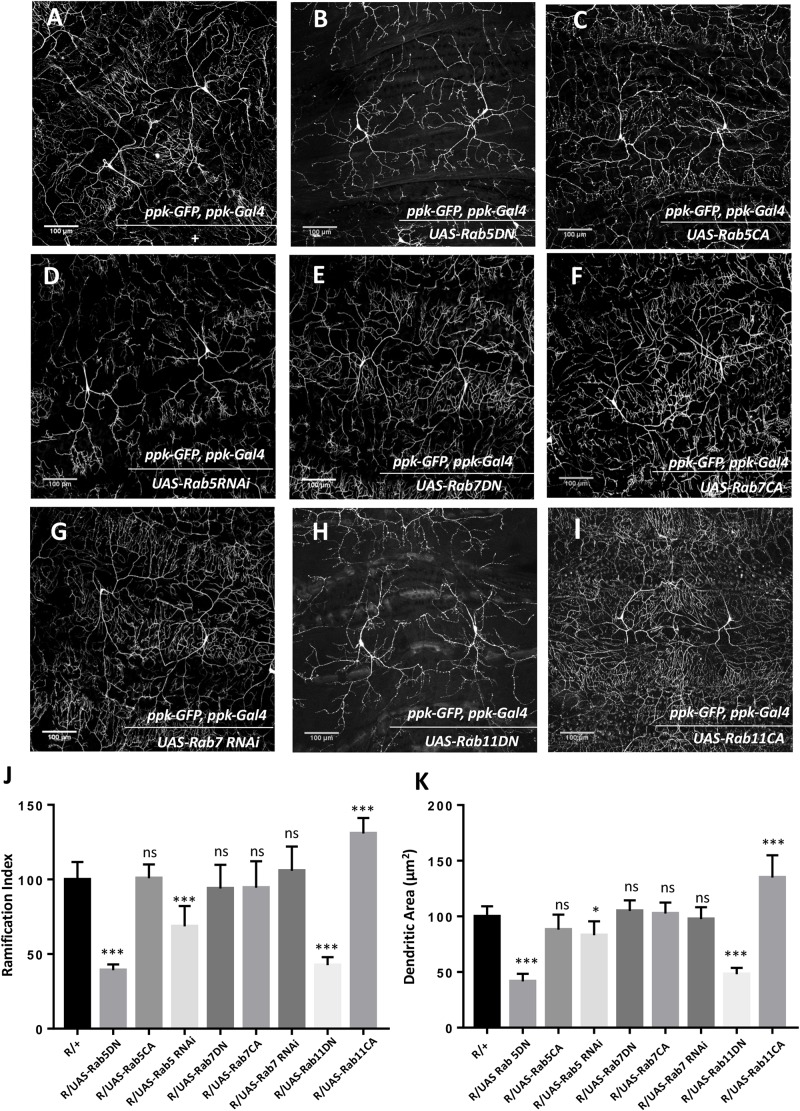
Rabs modulate dendritic arborization. **(A)** CIVda larvae imaged with the ‘wild-type’ reporter line (R/+: *ppk-GFP, ppk-Gal4*). Representative images are shown **(B–I)** for all experiments. **(B–D)** Rab5DN mutant expression or Rab5 knockdown using RNAi, reduces arborization (for Rab5 DN: *n* = 23, *N* = 4; for Rab5 RNAi: *n* = 40, *N* = 7) in Class IV neurons but Rab5CA mutant expression does not show any change (*n* = 18, *N* = 4), when compared to control R/+(*n* = 55, *N* = 5). **(E–G)** Rab7DN mutant expression, Rab7 knockdown using RNAi and Rab7CA mutant expression using *ppk-Gal4*, do not show any difference in arborization as compared to wild-type control (for Rab7DN: *n* = 35, *N* = 6; for Rab7 RNAi: *n* = 43, *N* = 7; for Rab7CA: *n* = 16, *N* = 4). **(H,I)** Rab11DN reduces arborization (*n* = 18, *N* = 4) when expressed in Class IV da neurons whereas Rab11CA mutant expression increases dendritic arbor complexity (*n* = 8, *N* = 3). **(J,K)** Quantitation of the extent of arborization in CIVDa using R.I and D.A. Values for D.L and D.BP are displayed in Supplementary Figure S2. ns, not significant. ^*^p < 0.05 and ^∗∗∗^*p* < 0.001. Error bars represent standard error. Statistical analysis using Dunnet’s multiple comparison test using GraphPad Prism 7 with exact *p*-values listed in Supplementary Table S1.

Given the role of Mon1 in endocytic trafficking, we sought to explore the role of Rab proteins in Mon1 mediated CIVda morphogenesis (Figure 2 and Supplementary Figures S2A,B). An earlier study in *Drosophila* has implicated Rab5 and the distribution of Rab5 endosomes in the patterning of da neurons (Satoh et al., 2008), while Rab11 mediated recycling has been shown to be important in dendritic branching in rat hippocampal neurons (Satoh et al., 2008; Lazo et al., 2013). In *Drosophila*, the roles of Rab11 mediated recycling pathway or the Rab7 mediated degradative pathway in dendrite morphology has not been tested. We therefore sought to test this in the context of Mon1 mutants through genetic epistasis, by using loss-of-function and gain-of-function transgenic lines against *Drosophila* Rab5, Rab7, and Rab11 genes. In agreement with earlier studies (Satoh et al., 2008), expression of Rab5 dominant negative (Rab5DN; Rab5 in a GDP-bound form) or knockdown using RNA interference, using *ppk-Gal4*, show a drastic reduction in the extent of arborization and branching (Figures 2B,D,J,K and Supplementary Figures S2A,B), with hypomorphic RNAi alleles demonstrating weaker effects. Our analysis indicated a 60–70% decrease in R.I, D.A, D.L, and D.BP for the Rab5DN allele. In contrast, the constitutively active (CA) form of Rab5 (RAB5CA, GTP-bound form) did not show any significant differences in R.I, D.A, and D.L (Figures 2C,J,K and Supplementary Figure S2B), while the D.BP were decreased by 20% (Supplementary Figure S2A) in CIVda as compared to the control (Figures 2A,J,K and Supplementary Figures S2A,B).

Cargo present in Rab5 positive early endosome cycle can be channeled down the degradation pathway involving Rab7 or the recycling pathway, marked by Rab11. We tested the involvement of these pathways by modulating the activity of Rab7 and Rab11. We found that increasing Rab7 activity through expression of a CA form, or decreasing Rab7 function by using a dominant negative (DN) form of Rab7 or through expression of Rab7 RNAi the *ppk* domain (Figures 2E,F,G,J,K and Supplementary Figures S2A,B) does not affect the arborization, branching, length or area of CIVda neurons. In contrast, expression of both, Rab11CA and Rab11DN altered arborization patterns in an opposing manner: increase in Rab11 activity increased R.I, D.A, D.L and D.BP, while a decrease in Rab11 activity reduced these parameters (Figures 2H–K and Supplementary Figures S2A,B). Interestingly, the increase in parameters (15–40%) seen upon expression of Rab11CA (Figures 2H–K) were correlated to and comparable with increase seen in Mon1 mutants, with the exception of D.BP, where Rab11CA has a weaker effect, suggesting that the Rab11 mediated recycling pathway plays a central role in CIVda patterning.

### Mon1 Interacts With Rabs to Modulate da

**FIGURE 3:**
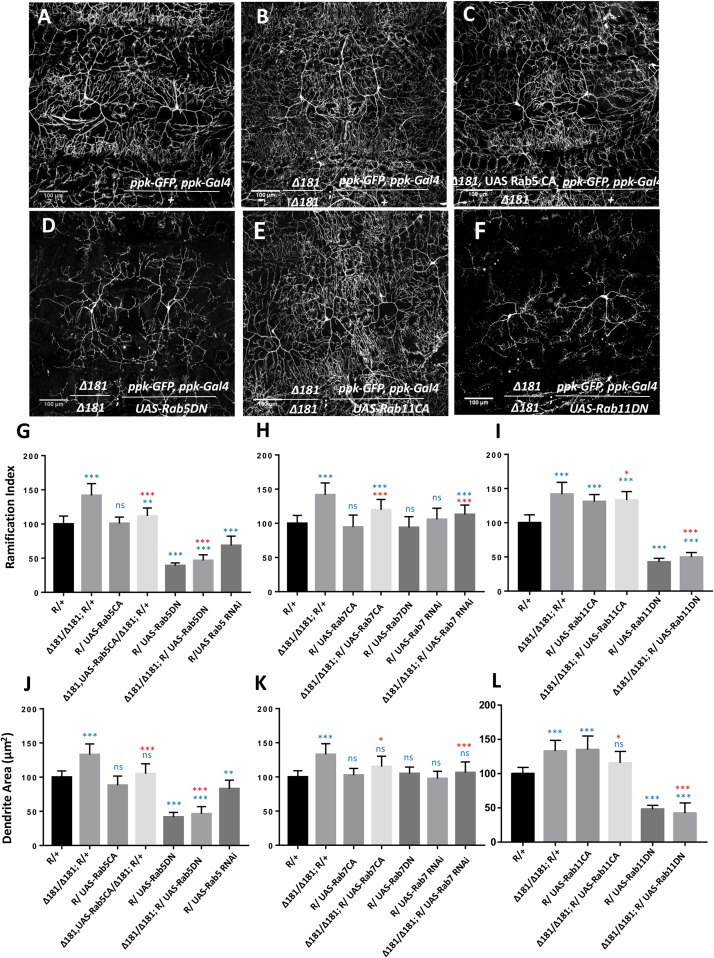
Mon1 interacts with Rabs to modulate dendritic arborization. **(A,B)** CIVda larvae imaged with the reporter line R/+ (*n* = 55, *N* = 7) and the *Dmon1^Δ181^/Dmon1^Δ181^; R/+* (*n* = 38, *N* = 7). Representative images are shown **(B–F)** for all experiments. For **(G–L)**, blue asterisk/n.s represents statistical comparison with wild-type (R/+) while red asterisk/n.s is a comparison with *Dmon1^Δ181^.* **(C,D)** Rab5CA expression in *Dmon1*^Δ181^ larvae shows significant reduction in parameters measured (R.I, D.A, D.L, D.BP) as compared to *Dmon1*^Δ181^ (*n* = 30, *N* = 7). Rab5DN in *Dmon1*^Δ181^ larvae shows reduction in arborization as compared to both *Dmon1*^Δ181^ (*n* = 26, *N* = 5) and wild-type larvae (*n* = 26, *N* = 5). Rab7CA expression in *Dmon1*^Δ181^ larvae shows reduction in arborization as compared to *Dmon1*^Δ181^ (*n* = 20, *N* = 4) and increase with respect to the wild-type (*n* = 20, *N* = 4). Rab7 RNAi in *Dmon1*^Δ181^ larave shows reduction in arborization as compared to *Dmon1*^Δ181^ (*n* = 43, *N* = 7) and increase with respect to the wild-type (*n* = 43, *N* = 7). Images for these experiments are not shown but data is quantified in **(H,K)**. **(E,F)** Rab11CA in *Dmon1*^Δ181^ background shows reduction in arborization as compared to *Dmon1*^Δ181^ larvae (*n* = 36, *N* = 6) and increase with respect to the wild-type larvae (*n* = 36, *N* = 6). Rab11 DN in *Dmon1*^Δ181^ larvae shows reduction in arborization as compared to *Dmon1*^Δ181^ (*n* = 19, *N* = 4) and decrease with respect to the control larvae (*n* = 19, *N* = 4). **(G,L)** Quantitation of the extent of arborization in CIVDa using R.I and D.A. Values for D.L and D.BP are displayed in Supplementary Figure S2. ns, not significant. ^*^p < 0.05, ^∗∗^*p* < 0.01, and ^∗∗∗^*p* < 0.001. Error bars represent standard error. Statistical analysis using Dunnet’s multiple comparison test using GraphPad Prism 7 with exact *p*-values listed in Supplementary Table S1.

Since the activity of Rab5 and Rab11 strongly modulates arborization of CIVda neurons, we explored the nature of the interaction between these Rabs 5, 7, and 11 and Mon1 to uncover features of vesicular recycling that are important for CIVda morphogenesis (Figure 3 and Supplementary Figures S2C–H). We tested this by modulating activity of Rab5, Rab7, and Rab11 in the *Dmon1* loss-of-function line (*Dmon1^Δ181^* or Δ181*)*, in combination with the reporter line (R) generated earlier (Figure 1A and see section “Materials and Methods”). Expression of Rab5CA in the *Dmon1*^Δ181^ larvae showed a partial rescue (Figures 3C,G,J and Supplementary Figures S2C,F) of the increase in R.I, D.A, D.L and D.BP, seen in the mutants, while expression of Rab5DN in the *Dmon1*^Δ181^ larvae led to a quantitative parameters (D.A., D.L, and D.BP) that were comparable to that of Rab5DN alone (Figures 3D,G,J and Supplementary Figures S2C,F).

Although Rab7 on its own does not seem to participate in the regulation of da (Figures 2F,G,J,K), we found that expression of Rab7CA leads to a significant reduction of R.I, D.A., D.L and D.BP, as compared to control *Dmon1*^Δ181^ larvae (Figures 3H–K and Supplementary Figures S2D,G; Images not displayed) suggesting that activation of the downstream endo-lysosomal pathway can suppress excess arborization in the mutant. This may be an outcome of a flux change due to modulation of the lysosomal branch. Interestingly, Rab7 RNAi also suppresses Mon1 loss of function phenotype (Figures 3H,K and Supplementary Figures S2D,G; Images not displayed), again suggesting change in flux of recycling vesicular trafficking in response to modulation of the lysosomal pathway. Expression of Rab11CA in *Dmon1*^Δ181^ larvae (weakly) rescues all measured parameters (R.I, D.A, D.L) except D.BP. Expression of Rab11DN, with or without *Dmon1*^Δ181^ in the background leads to lower values (decrease of 60–70%) of R.I, D.A., D.L, and D.BP.** This lends support to the view that the Rab11 mediated re-cycling pathway functions downstream of Mon1 and may play an important role in manifestation of the arborization defect in *Dmon1*^Δ181^ mutants.

## Discussion

**FIGURE 4:**
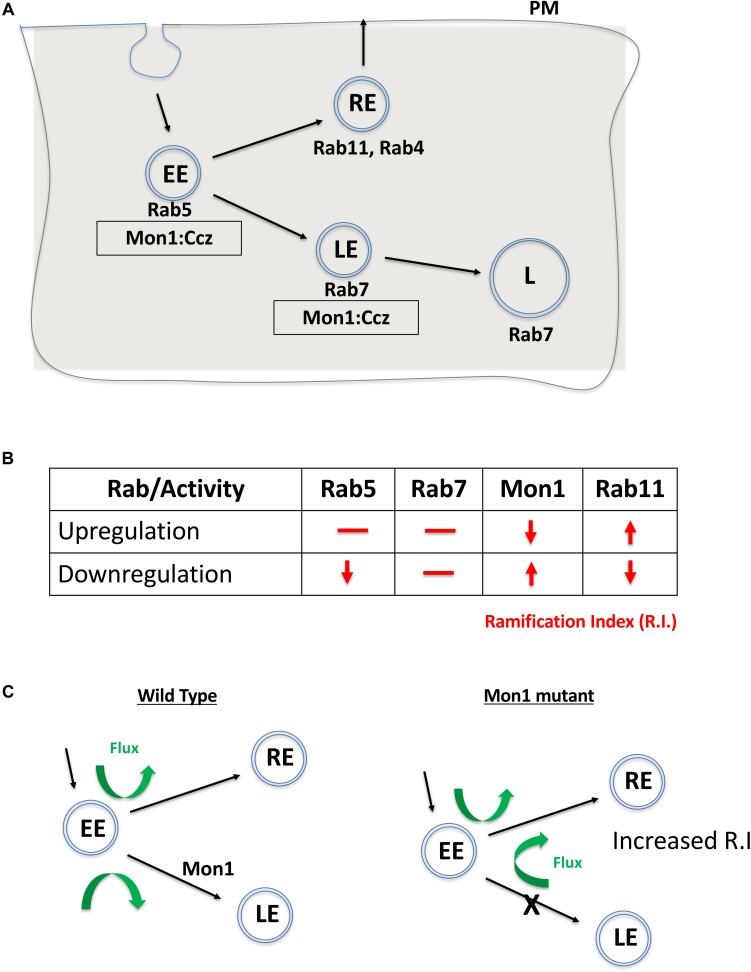
Mon1 levels may regulate vesicular flux through the recycling pathway. **(A)** Schematic shows the endocytic pathway branching at the early endosome, with vesicles entering either the degradative or recycling pathways. Rab5 marks early endosomes (EE), Rab7 marks late endosomes (LE) while Rab11 marks the recycling endosome (RE). The Mon1:Ccz complex acts as a GEF for conversion of EE to LE. **(B)** Effect of upregulation or downregulation of Rab or Mon1 activity on complexity of dendritic arborisation, as measured by change in R.I (red lines/arrows). R.I decreases with decrease in Rab5 and Rab11 activity as also with increase of Mon1 function. In contrast, R.I increases with decrease in Mon1 function and increase in Rab11 activity. Similar trends are seen in D.A, D.L, and D.BP. **(C)** A hypothetical model, that agrees with our data, is the requirement of endocytic recycling for increase in dendritic arborization. Thus, increase in RE endosomal flux may influence branching. This can be done directly by increasing RE by overexpressing Rab11, or indirectly by increasing the endocytic flux to the RE pathway, either by decreasing Mon1 function or by decreasing Rab5 activity. The proposed model relies on a minimal role for Rab7 in regulating R.I. Green arrows depict the increase in flux of vesicular trafficking in the RE pathway when Mon1 function is reduced.

Mon1 is a conserved eukaryotic protein with a ‘longin’ domain. The domain has an alpha-beta-alpha sandwich architecture and is a feature of endocytic trafficking proteins (De Franceschi et al., 2014). Protein containing ‘longin’ domains include SANDs, SNAREs, targetins, adaptins, and sedlins (De Franceschi et al., 2014). The dimeric Mon1:CCZ complex is involved in Rab conversion and is a GEF for Rab7 (Figure 4A). Additionally, there is evidence that Mon1 can be secreted by neurons, either in membrane bound or unbound form (Deivasigamani et al., 2015). Mon1 may thus regulate anterograde signaling in synapses, both neuron:neuron or neuron:muscle. Recent research from our laboratory (Dhiman et al., 2019) also suggests that Mon1 in Octopaminergic neurons regulates systemic insulin signaling by regulating insulin producing cells.

CIVda neurons are sensitive to the dose of DMon1 in the cell (Figure 4B). Decrease in *Dmon1* leads to increased complexity of arborization, which includes increase in branching, length and area covered by the axons. Increase in *Dmon1* decreases complexity of arborization and reduces the values of the parameters measured. Since Mon1 is primarily known for its role as a Rab converter in eukaryotic cells, we explored functions of Rab proteins Rab5, Rab7, and Rab11 in regulating da by themselves and also in the context of *Dmon1* loss of function. Amongst the Rabs tested, Rab7 activity did not appear to affect dendritic morphology, suggesting that perturbation of late endosomal trafficking does not affect morphogenesis of CIVda neurons in larvae. This premise however can be strengthened by using Mosaic analysis with a repressible cell marker, studies. In contrast, Rab11 activity altered arbor complexity with decrease in Rab11 activity leading to a dramatic decrease in arborization and expression of Rab11CA having an opposite effect (Figure 3). This suggests that recycling endosomes play critical roles in determining arbor complexity which is in agreement with the role of Rab11 in dendrite morphogenesis in vertebrate systems (Villarroel-Campos et al., 2014; Peng et al., 2015; Gu et al., 2016). Since reduction of Rab5 activity also decreases arbor complexity, and Rab5 ‘sorting’ endosomes are upstream (Figure 4A) of both recycling endosomes (RE) and late endosomes (LE), we propose that vesicular flux through the RE but not the LE is a central determinant of CIVda morphogenesis (Figure 4C). Loss of Rab5 leads to a decrease in the rate of RE formation, while loss of DMon1 changes the endocytic flux, shunting excess early endosomes (EE’s) toward the RE pathway. In our model (Figure 4C), increase in activity of Mon1 enhances Rab5 to Rab7 conversion, reducing RE traffic leading to decreased arborization.

How does increase in RE flux lead to increase in dendritic complexity? Since endosomes marked with Rab11 can be exocytosed, we suggest that enhanced vesicular flux in the RE pathway leads to enhanced exocytosis, which in turn is correlated to increase dendritic arborization. This is in agreement with earlier studies where trafficking of cargo in Rab11 vesicles regulated dendritic complexity. For example, Lazo et al. (2013) demonstrate that Rab11 regulates trafficking of brain derived neurotrophic factor along with its receptor TrkB while Peng et al. (2015) have shown that the Rop-exocyst complex is important for dendritic branching in CIVda neurons. In contrast, data from the Klein lab (Yousefian et al., 2013), with experiments performed in the wing imaginal disk, suggest that Rab11 is not affected in the *Dmon1*^*mut4*^ lines, and instead find changes in the Rab4 associated fast recycling pathway. In addition to protein and RNA based cargo, exocytosis also provides neuronal membrane that is critical for growth of the arbor, underscoring the importance of RE flux and exocytosis.

The balance of endocytosis and exocytosis is crucial for the growth and maintenance of CIVda arbors. Mon1 activity in the early ‘sorting’ endosomes may be important for modulating the flux through either the LE or RE pathways which in turn could lead to modulation of neuronal architecture. Our results thus underscore the role of endocytic flux in dendrite morphogenesis. The genetic interactions described here suggest a cell-autonomous role for Mon1. Given the ability of Mon1 to be secreted, it would be interesting, in future studies, to test for possible non-autonomous roles in dendrite development.

## Materials and Methods

### Fly Husbandry

Fly lines were maintained at 25^∘^C on standard cornmeal agar medium. UAS-GAL4 system (Brand and Perrimon, 1993) was used for over-expression of transgenes.

### Transgenic Lines

*Dmon1*^Δ181^ was generated earlier through excision of *pUAST-Rab21:: YFP* insertion (Deivasigamani et al., 2015). Lines procured through the Bloomington *Drosophila* Stock Centre include Df(2L)9062, 35843 (*ppk-GFP*), 32079 (*ppk-Gal4*), 9772 (*UAS-Rab5-DN*), 9773 (*Rab5CA*), 34832 (*Rab5 RNAi*), 9779 (*UAS*-*YFP:Rab7CA*), 9778 (*UAS-Rab7DN*), 27051 (*UAS-Rab7 RNAi*), 9792 (*UAS-Rab11DN*), 9791 (*UAS-Rab11CA*). *UAS-Mon1:HA/TM6Tb* is a kind gift from Prof. Thomas Klein.

### Immunohistochemistry and Imaging

Wandering third instar larvae were collected, fileted in 1X PBS, dissected and fixed in Bouins solution (HT10132, Sigma) for 7 min. The tissues were then blocked in 1X PBS +0.3%Triton (194854, MP Biomedicals) and 2% BSA stained overnight in anti-GFP (Chk, A10262, Invitrogen, 1:500 and Rb, A6455, 1:1000) incubated in 4^∘^C overnight. This was followed by washes with 1X PBS +0.3%Triton and 2% Triton-X and incubated in secondary antibody at room temperature for 1.5 h. The final step involved washes and mounting of tissues in 70% glycerol with n-propyl gallate. Confocal imaging was carried out using a Zeiss LSM 710 microscope at 20X magnification. For Rab staining, dilutions used were as follows. Anti Rab5 (1:500), Rab7(1:500), Rab11(1:500) (Tanaka and Nakamura, 2008). Fixative used for staining with Rab antibodies was 4% PFA. Secondaries used were, Rab5 (1:1000 Guinea Pig Alexa fluor 568, Invitrogen A11075), Rab7 and Rab11 (1:1000 Rabbit Alexa Fluor 647, Invitrogen A21244), Chicken Alexa Fluor 488 from Invitrogen (A11039) and Rabbit Alexa Fluor 488 from Invitrogen (A11034). Rab antibodies were a kind gift by Prof. Akira Nakamura (RIKEN Center for Developmental Biology, Kobe, Japan). For Rabs, confocal imaging was carried out using a Zeiss LSM 710 microscope at 63X magnification.

### Sholl Analysis

Sholl analysis, to measure the ramification index (R.I), was performed using the NIH ImageJ Sholl Analysis Plugin (v1.0)^1^, as distributed by Fiji. Briefly, the maximum intensity projection for z stacks of each neuron was converted to a segmented grayscale image using ImageJ. Background dendrites extending into the image view from neighboring neurons were manually deleted. Sholl analysis was performed by drawing a straight line from the cell body to the distal tip of the neuron. The area for the analysis was hence defined by this straight line which is considered as the radii for each image. The origin of the concentric radii was set at the midpoint of the longest axis of the soma. Analysis was performed in automated way using the following parameters: starting radius, 1 μm; radius step size, 2 μm; span, 1 μm; span type, median. The number of dendrite intersections for each circle is measured and the highest value is divided by the number of primary dendrites (intersections at the starting radius) to obtain the Schoen ramification index (R.I). This parameter is dependent on maximum number of intersections and the number of primary dendrites. Statistics were performed using the Prism statistical package (GraphPad, San Diego, CA, United States).

### Neuromorphometric Analysis of da Neurons

The Filament dendrite tracer plug-in of the IMARIS 7 software was used to trace the dendrites in 3D and generate quantitative data for each genotype. The analysis was performed on dendritic arbor’s arising from a single neuron and the branches from neighboring neurons were deleted manually. The cell body was defined as the origin by adjusting the threshold for the largest diameter. The ‘Dendritic Area (D.A)’ measured in μm^2^ refers to the total area occupied by the traced filament. The ‘Dendritic length (D.L),’ measured in μm refers to the sum of all filaments traced within an arbor. The total number of ‘Dendritic Branch points (D.BP)’ in each arbor have also been measured for each genotype. The numbers for D.BP are the sum of all branch points in the image (primary, secondary, and tertiary).

## Data Availability

All datasets generated for this study are included in the manuscript and/or the Supplementary Files.

## Ethics Statement

This study was carried out in accordance with the recommendations of the IISER Institutional Biosafety Committee (IBSC). The IBSC is a statutory committee established by the Department of Biotechnology, Government of India and has approved the protocols used in our study.

## Author Contributions

SD, RKH, AR, and GR conceived the project and designed the experiments. RKH, ST, and SD performed all the experiments. RKH, SD, AR, ST, and GR analyzed the data and wrote the manuscript.

## Conflict of Interest Statement

The authors declare that the research was conducted in the absence of any commercial or financial relationships that could be construed as a potential conflict of interest.

## Footnotes

1 https://imagej.net/Sholl_Analysis

## Supplementary Material

FIGURE S1 Antibody staining of cell body of CIVDa neurons with antibodies against GFP, Rab5, Rab7, and Rab11. **(A)** Control larvae with the reporter (R: ppk-GFP, ppk-Gal4) show localization of Rab5 and Rab7 as distinct punctate. **(B)** *Dmon1*^Δ181^/*Dmon1*^Δ181^ displays enhanced accumulation of Rab5 as compared to the control, while the Rab7 does not show localization in endosomes. **(C)** Control larvae with the reporter (R: ppk-GFP, ppk-Gal4) show localization of Rab5 and Rab 11 as distinct punctate. **(D)** *Dmon1*^Δ181^/*Dmon1*^Δ181^ mutant shows enhanced accumulation of Rab5, as compared to control, with increase in punctae. Rab11 punctae do not appear to change significantly.

FIGURE S2 Quantitative parameters (D.BP, D.L, and D.A) for experiments displayed in main Figures 1–3. For all panels, blue asterisk/ns represents statistical comparison with wild-type (R/+) while red asterisk/ns is a comparison with *Dmon1*^Δ181^. **(A)** Graph represents normalized values that appear to follow similar trend as that of the R.I (Figure 2). Statistical values for the genotypes when expressed in the ppk-Gal4 domain in wild-type animals: Rab5CA expression (*N* = 4, *p* = 0.0073), Rab5DN expression (*N* = 4, *p* = 0.0001), Rab5 knockdown using RNAi (*N* = 4, *p* = 0.0002), Rab7CA expression (*N* = 4, *p* = 0.9999), Rab7DN expression (*N* = 4, *p* = 0.9997), Rab7 knockdown using RNAi (*N* = 4, *p* = 0.9999), Rab11CA expression (*N* = 4, *p* = 0.0329), Rab11DN expression (*N* = 4, *p* = 0.0001). n(number of neurons) analyzed for each genotype is 10. **(B)** Quantitation of (total) dendritic length (D.L). Statistical Values for the genotypes when expressed using ppk-Gal4 in wild-type background are as follows: Rab5CA expression (*N* = 4, *p* = 0.3142), Rab5DN expression (*N* = 4, *p* = 0.0001), Rab5 knockdown using RNAi (*N* = 4, *p* = 0.0164), Rab7CA expression (*N* = 4, *p* = 0.9991), Rab7DN expression (*N* = 4, *p* = 0.9754), Rab7 knockdown using RNAi (*N* = 4, *p* = 0.9996), Rab11 expression (*N* = 4, *p* = 0.0329), Rab11DN expression (*N* = 4, *p* = 0.0001). **(C–E)** Quantification of number of Dendrite Branch points (D.BP) for the interaction of Mon1 with different Rabs. Statistical values (blue ^*^’s or ns), for the genotypes when expressed using ppk-Gal4 in *Dmon1*^Δ181^ background as compared to control alone are as follows: Rab5CA expression (*N* = 4, *p* = 0.9990), Rab5DN expression (*N* = 4, *p* = 0.0001), Rab7CA expression (*N* = 4, *p* = 0.0019), Rab7 knockdown using RNAi (*N* = 4, *p* = 0.0992), Rab11CA expression (*N* = 4, *p* = 0.0001), Rab11DN expression (*N* = 4, *p* = 0.0001). Statistical values (red ^*^’s or ns) for the genotypes when expressed with ppk-Gal4 in *Dmon1*^Δ181^ background as compared to *Dmon1*^Δ181^ are as follows: Rab5CA expression (*N* = 4, *p* = 0.0002), Rab5DN expression (*N* = 4, *p* = 0.0001), Rab7CA expression (*N* = 4, *p* = 0.8603), Rab7 knockdown using RNAi (*N* = 4, *p* = 0.1021), Rab11CA expression (*N* = 4, *p* = 0.9999), Rab11DN expression (*N* = 4, *p* = 0.0001). **(F–H)** Quantification of Dendrite Length (D.L) for the interaction of Mon1 with different Rabs. The graph represents normalized values which follow similar trend as that of the R.I (Figure 3). Statistical values (blue ^*^’s or ns) for each of the genotypes when expressed in the ppk-Gal4 domain in *Dmon1*^Δ181^ background as compared to control alone are as follows: Rab5CA expression (*N* = 4, *p* = 0.9997), Rab5DN expression (*N* = 4, *p* = 0.0001), Rab7CA expression (*N* = 4, *p* = 0.0125), Rab7 knockdown using RNAi (*N* = 4, *p* = 0.7656), Rab11CA expression (*N* = 4, *p* = 0.7089), Rab11DN expression (*N* = 4, *p* = 0.0001). Statistical values (red ^*^’s or ns) for the genotypes when expressed using ppk-Gal4 in *Dmon1*^Δ181^ background as compared to *Dmon1*^Δ181^ are as follows: Rab5CA mutant overexpression (*N* = 4, *p* = 0.0001), Rab5DN expression (*N* = 4, *p* = 0.0001), Rab7CA expression (*N* = 4, *p* = 0.1130), Rab7 knockdown using RNAi (*N* = 4, *p* = 0.0002), Rab11CA expression (*N* = 4, *p* = 0.0003), Rab11DN expression (*N* = 4, *p* = 0.0001). Number of neurons analyzed for all genotypes = 10, ns, not significant. ^*^*p* < 0.05, ^∗∗^*p* < 0.01, ^∗∗∗^*p* < 0.001. Error bars represent standard error. *p-*Values generated using Dunnet’s multiple comparison test using GraphPad Prism 7.

TABLE S1 *p*-values for analysis of experiments conducted for this study.
