# Supplementary material for: Monensin Sensitive 1 Regulates Dendritic Arborization in Drosophila by Modulating Endocytic Flux
Source: Front Cell Dev Biol. 2019 Aug 2;7:145. doi: 10.3389/fcell.2019.00145 (PMC6687774; doi:10.3389/fcell.2019.00145)
Supplement: FIGURE S1 — Antibody staining of cell body of CIVDa neurons with antibodies against GFP, Rab5, Rab7, and Rab11. (A) Control larvae with the reporter (R: ppk-GFP, ppk-Gal4) show localization of Rab5 and Rab7 as distinct punctate. (B) Dmon1Δ181/Dmon1Δ181 displays enhanced accumulation of Rab5 as compared to the control, while the Rab7 does not show localization in endosomes. (C) Control larvae with the reporter (R: ppk-GFP, ppk-Gal4) show localization of Rab5 and Rab 11 as distinct punctate. (D) Dmon1Δ181/Dmon1Δ181 mutant shows enhanced accumulation of Rab5, as compared to control, with increase in punctae. Rab11 punctae do not appear to change significantly. [file Data_Sheet_1.PDF]

Suppl. Figure 1

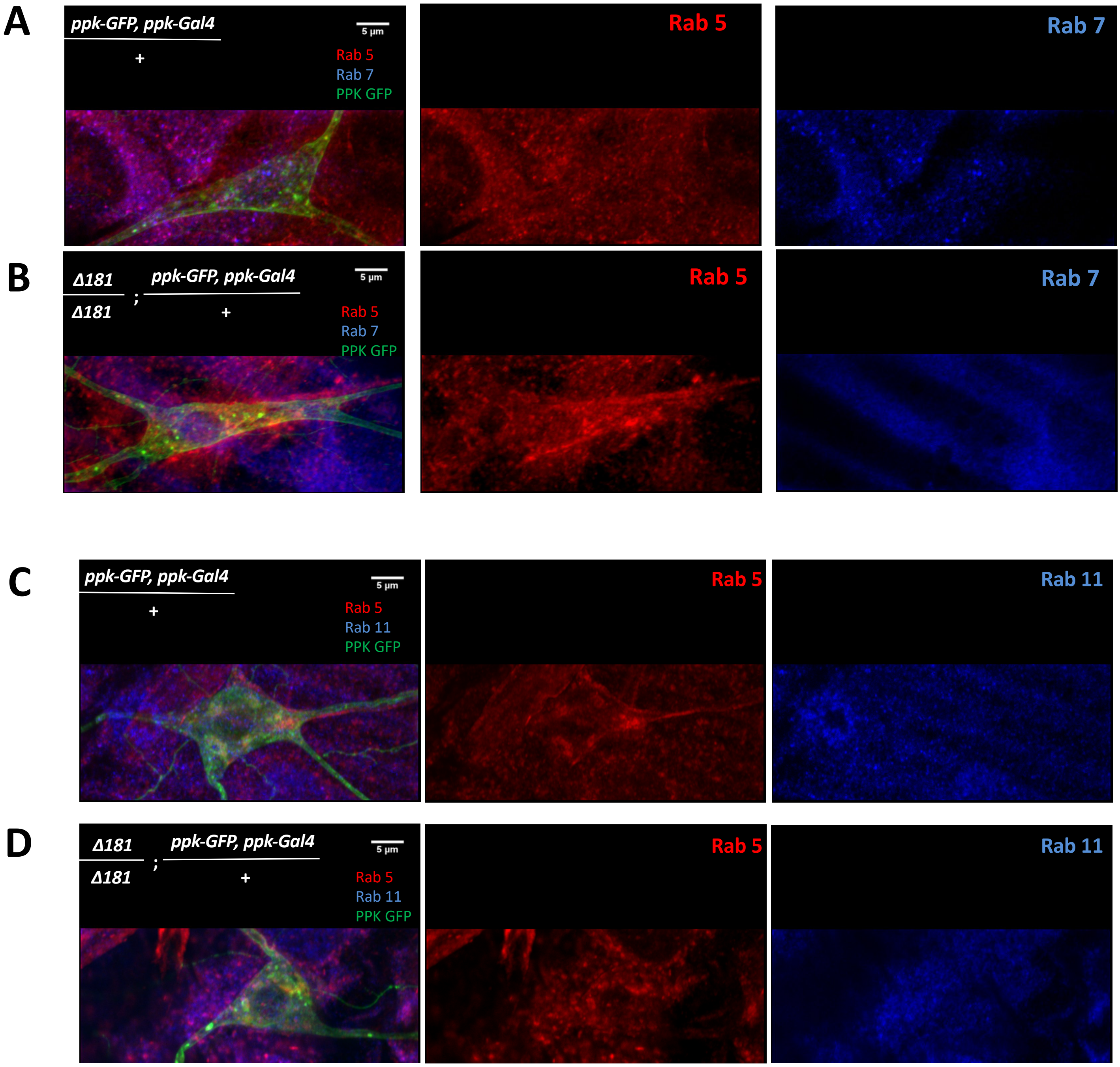

Supplementary Figure 1. Antibody staining of cell body of CIVDa neurons with antibodies against GFP, Rab5, Rab7 and Rab11.

A. Control larvae with the reporter (R: *ppk-GFP, ppk-Gal4*) show localization of Rab5 and Rab7 as distinct punctate.

B. $Dmon1^{\Delta181}/Dmon1^{\Delta181}$ displays enhanced accumulation of Rab5 as compared to the control, while the Rab7 does not show localization in endosomes.

C. Control larvae with the reporter (R: *ppk-GFP, ppk-Gal4*) show localization of Rab5 and Rab 11 as distinct punctate.

D. $Dmon1^{\Delta181}/Dmon1^{\Delta181}$ mutant shows enhanced accumulation of Rab5, as compared to control, with increase in punctae. Rab11 punctae do not appear to change significantly.
