# Supplementary material for: Monensin Sensitive 1 Regulates Dendritic Arborization in Drosophila by Modulating Endocytic Flux
Source: Front Cell Dev Biol. 2019 Aug 2;7:145. doi: 10.3389/fcell.2019.00145 (PMC6687774; doi:10.3389/fcell.2019.00145)
Supplement: FIGURE S2 — Quantitative parameters (D.BP, D.L, and D.A) for experiments displayed in main Figures 1–3. For all panels, blue asterisk/ns represents statistical comparison with wild-type (R/+) while red asterisk/ns is a comparison with Dmon1Δ181. (A) Graph represents normalized values that appear to follow similar trend as that of the R.I (Figure 2). Statistical values for the genotypes when expressed in the ppk-Gal4 domain in wild-type animals: Rab5CA expression (N = 4, p = 0.0073), Rab5DN expression (N = 4, p = 0.0001), Rab5 knockdown using RNAi (N = 4, p = 0.0002), Rab7CA expression (N = 4, p = 0.9999), Rab7DN expression (N = 4, p = 0.9997), Rab7 knockdown using RNAi (N = 4, p = 0.9999), Rab11CA expression (N = 4, p = 0.0329), Rab11DN expression (N = 4, p = 0.0001). n(number of neurons) analyzed for each genotype is 10. (B) Quantitation of (total) dendritic length (D.L). Statistical Values for the genotypes when expressed using ppk-Gal4 in wild-type background are as follows: Rab5CA expression (N = 4, p = 0.3142), Rab5DN expression (N = 4, p = 0.0001), Rab5 knockdown using RNAi (N = 4, p = 0.0164), Rab7CA expression (N = 4, p = 0.9991), Rab7DN expression (N = 4, p = 0.9754), Rab7 knockdown using RNAi (N = 4, p = 0.9996), Rab11 expression (N = 4, p = 0.0329), Rab11DN expression (N = 4, p = 0.0001). (C–E) Quantification of number of Dendrite Branch points (D.BP) for the interaction of Mon1 with different Rabs. Statistical values (blue *’s or ns), for the genotypes when expressed using ppk-Gal4 in Dmon1Δ181 background as compared to control alone are as follows: Rab5CA expression (N = 4, p = 0.9990), Rab5DN expression (N = 4, p = 0.0001), Rab7CA expression (N = 4, p = 0.0019), Rab7 knockdown using RNAi (N = 4, p = 0.0992), Rab11CA expression (N = 4, p = 0.0001), Rab11DN expression (N = 4, p = 0.0001). Statistical values (red *’s or ns) for the genotypes when expressed with ppk-Gal4 in Dmon1Δ181 background as compared to Dmon1Δ181 are as follows: Rab5CA expression (N = 4, p = 0.0 [file Data_Sheet_2.PDF]

Suppl. Figure 2

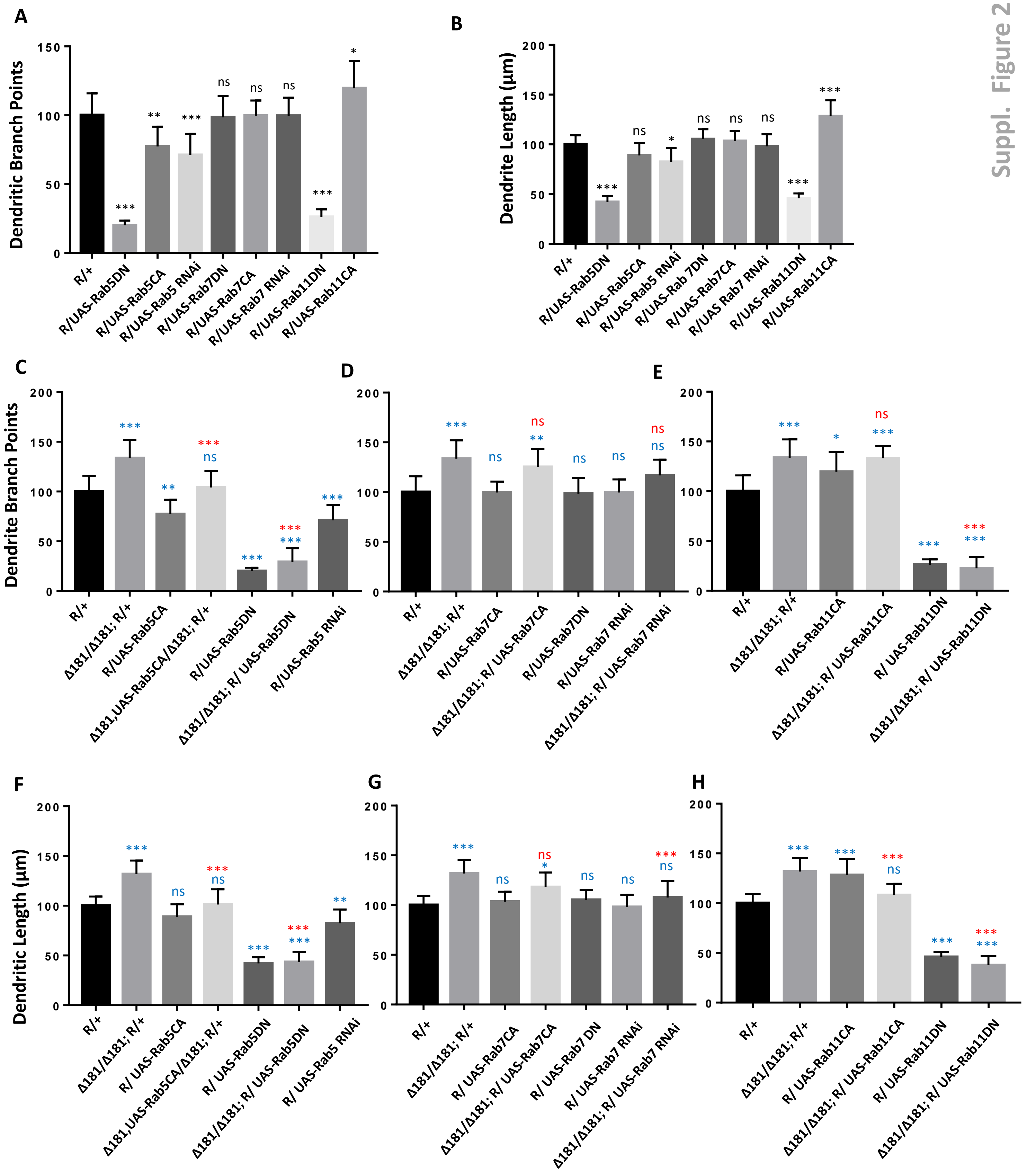

Supplementary Figure 2. Quantitative parameters (D.BP, D.L, and D.A) for experiments displayed in main Figures (1-3)

For all panels, blue asterix/ns represents statistical comparison with wild type (R/+) while red asterix/ns is a comparison with Dmon1$^{\Delta 181}$.

A. Graph represents normalized values that appear to follow similar trend as that of the R.I.(Fig 2). Statistical values for the genotypes when expressed in the *ppk-Gal4* domain in wild type animals: Rab5CA expression (N= 4, p=0.0073), Rab5DNexpression (N= 4, p=0.0001), Rab5 knockdown using RNAi (N= 4, p=0.0002), Rab7C expression (N= 4, p=0.9999), Rab7DN expression (N= 4, p=0.9997), Rab7 knockdown using RNAi (N= 4, p=0.9999), Rab11CA expression (N= 4, p=0.0329), Rab11DN expression (N= 4, p=0.0001). n(number of neurons) analysed for each genotype is 10.

B. Quantitation of (total) dendritic length (D.L). Statistical Values for the genotypes when expressed using *ppk-Gal4* in wild type background are as follows: Rab5CA expression (N= 4, p=0.3142), Rab5DN expression (N= 4, p=0.0001), Rab5 knockdown using RNAi (N= 4, p=0.0164), Rab7CA expression (N= 4, p=0.9991), Rab7DNexpression (N= 4, p=0.9754), Rab7 knockdown using RNAi (N= 4, p=0.9996), Rab11expression (N= 4, p=0.0329), Rab11DN expression (N= 4, p=0.0001).

C-E. Quantification of number of Dendrite Branch points (D.BP) for the interaction of *Mon1* with different *Rabs*. Statistical values (blue *'s or ns), for the genotypes when expressed using *ppk-Gal4* in $Dmon1^{\Delta 181}$ background as compared to control alone are as follows: Rab5CA expression(N= 4, p=0.9990), Rab5DN expression (N= 4, p=0.0001), Rab7CA expression (N= 4, p=0.0019), Rab7 knockdown using RNAi (N= 4, p=0.0992), Rab11CA expression (N= 4, p=0.0001), Rab11 DN expression (N= 4, p=0.0001).
Statistical values (red *'s or ns) for the genotypes when expressed with *ppk-Gal4* in $Dmon1^{\Delta 181}$ background as compared to $Dmon1^{\Delta 181}$ are as follows: Rab5CA expression(N= 4, p=0.0002), Rab5DN expression (N= 4, p=0.0001), Rab7CA expression (N= 4, p=0.8603), Rab7 knockdown using RNAi (N= 4, p=0.1021), Rab11CA expression (N= 4, p=0.9999), Rab11DN expression (N= 4, p=0.0001).

F-H. Quantification of Dendrite Length (D.L) for the interaction of *Mon1* with different *Rabs*. The graph represents normalized values which follow similar trend as that of the R.I.(Fig. 3). Statistical values (blue *'s or ns) for each of the genotypes when expressed in the *ppk-Gal4* domain in $Dmon1^{\Delta 181}$ background as compared to control alone are as follows: Rab5CA expression(N= 4, p=0.9997), Rab5DN expression (N= 4, p=0.0001), Rab7CA expression (N= 4, p=0.0125), Rab7 knockdown using RNAi (N= 4, p=0.7656), Rab11CA expression (N= 4, p=0.7089), Rab11DN expression (N= 4, p=0.0001).
Statistical values (red *'s or ns) for the genotypes when expressed using *ppk-Gal4* in $Dmon1^{\Delta 181}$ background as compared to $Dmon1^{\Delta 181}$ are as follows: Rab5CA mutant overexpression(N= 4, p=0.0001), Rab5DN expression (N= 4, p=0.0001), Rab7CA expression (N= 4, p=0.1130), Rab7 knockdown using RNAi (N= 4, p=0.0002), Rab11CA expression (N= 4, p=0.0003), Rab11DN expression (N= 4, p=0.0001).

Note: Number of neurons analysed for all genotypes= 10, ns: not significant,. ***p<0.001, **p<0.01, *p<0.05 Error bars represent standard error. p values generated using Dunnet's multiple comparison test using Graphpad Prism 7.
